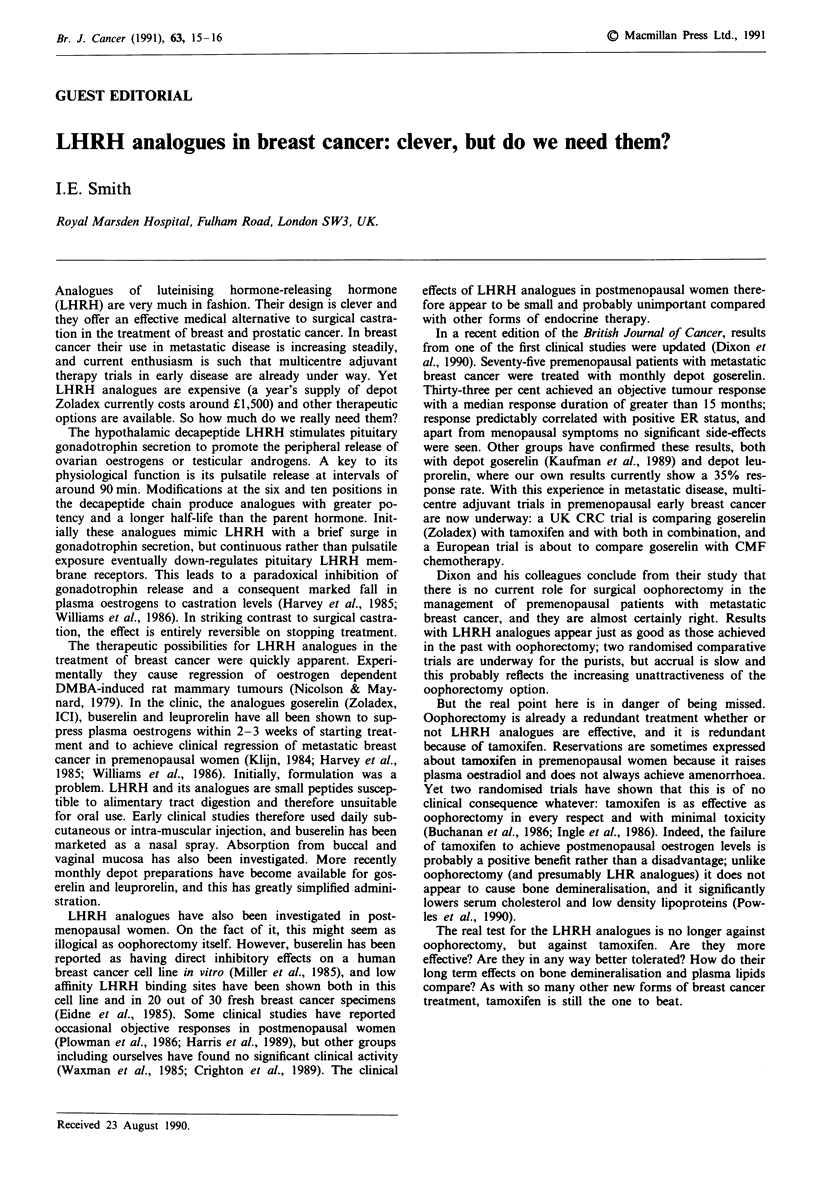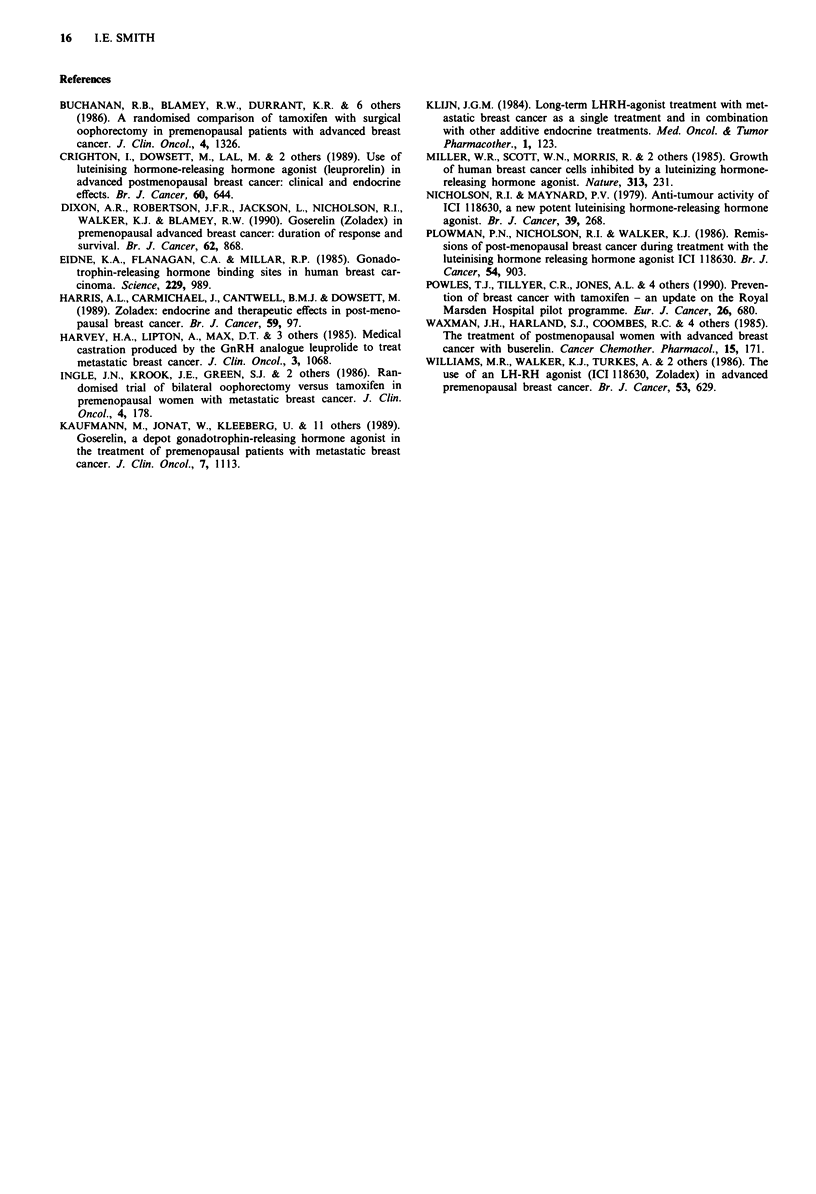# LHRH analogues in breast cancer: clever, but do we need them?

**DOI:** 10.1038/bjc.1991.6

**Published:** 1991-01

**Authors:** I. E. Smith


Br. J. Cancer (1991), 63, 15-16 © Macmillan Press Ltd., 1991

GUEST EDITORIAL

### LHRH analogues in breast cancer: clever, but do we need them?

I.E. Smith

*Royal Marsden Hospital, Fulham Road, London SW3, UK.*

Analogues of luteinising hormone-releasing hormone (LHRH) are very much in fashion. Their design is clever and they offer an effective medical alternative to surgical castration in the treatment of breast and prostatic cancer. In breast cancer their use in metastatic disease is increasing steadily, and current enthusiasm is such that multicentre adjuvant therapy trials in early disease are already under way. Yet LHRH analogues are expensive (a year's supply of depot Zoladex currently costs around £1,500) and other therapeutic options are available. So how much do we really need them?

The hypothalamic decapeptide LHRH stimulates pituitary gonadotrophin secretion to promote the peripheral release of ovarian oestrogens or testicular androgens. A key to its physiological function is its pulsatile release at intervals of around 90 min. Modifications at the six and ten positions in the decapeptide chain produce analogues with greater potency and a longer half-life than the parent hormone. Initially these analogues mimic LHRH with a brief surge in gonadotrophin secretion, but continuous rather than pulsatile exposure eventually down-regulates pituitary LHRH membrane receptors. This leads to a paradoxical inhibition of gonadotrophin release and a consequent marked fall in plasma oestrogens to castration levels (Harvey *et al.*, 1985; Williams *et al.*, 1986). In striking contrast to surgical castration, the effect is entirely reversible on stopping treatment.

The therapeutic possibilities for LHRH analogues in the treatment of breast cancer were quickly apparent. Experimentally they cause regression of oestrogen dependent DMBA-induced rat mammary tumours (Nicolson & Maynard, 1979). In the clinic, the analogues goserelin (Zoladex, ICI), buserelin and leuprorelin have all been shown to suppress plasma oestrogens within 2-3 weeks of starting treatment and to achieve clinical regression of metastatic breast cancer in premenopausal women (Klijn, 1984; Harvey *et al.*, 1985; Williams *et al.*, 1986). Initially, formulation was a problem. LHRH and its analogues are small peptides susceptible to alimentary tract digestion and therefore unsuitable for oral use. Early clinical studies therefore used daily subcutaneous or intra-muscular injection, and buserelin has been marketed as a nasal spray. Absorption from buccal and vaginal mucosa has also been investigated. More recently monthly depot preparations have become available for goserelin and leuprorelin, and this has greatly simplified administration.

LHRH analogues have also been investigated in postmenopausal women. On the fact of it, this might seem as illogical as oophorectomy itself. However, buserelin has been reported as having direct inhibitory effects on a human breast cancer cell line *in vitro* (Miller *et al.*, 1985), and low affinity LHRH binding sites have been shown both in this cell line and in 20 out of 30 fresh breast cancer specimens (Eidne *et al.*, 1985). Some clinical studies have reported occasional objective responses in postmenopausal women (Plowman *et al.*, 1986; Harris *et al.*, 1989), but other groups including ourselves have found no significant clinical activity (Waxman *et al.*, 1985; Crighton *et al.*, 1989). The clinical effects of LHRH analogues in postmenopausal women therefore appear to be small and probably unimportant compared with other forms of endocrine therapy.

In a recent edition of the *British Journal of Cancer*, results from one of the first clinical studies were updated (Dixon *et al.*, 1990). Seventy-five premenopausal patients with metastatic breast cancer were treated with monthly depot goserelin. Thirty-three per cent achieved an objective tumour response with a median response duration of greater than 15 months; response predictably correlated with positive ER status, and apart from menopausal symptoms no significant side-effects were seen. Other groups have confirmed these results, both with depot goserelin (Kaufman *et al.*, 1989) and depot leuprorelin, where our own results currently show a 35% response rate. With this experience in metastatic disease, multicentre adjuvant trials in premenopausal early breast cancer are now underway: a UK CRC trial is comparing goserelin (Zoladex) with tamoxifen and with both in combination, and a European trial is about to compare goserelin with CMF chemotherapy.

Dixon and his colleagues conclude from their study that there is no current role for surgical oophorectomy in the management of premenopausal patients with metastatic breast cancer, and they are almost certainly right. Results with LHRH analogues appear just as good as those achieved in the past with oophorectomy; two randomised comparative trials are underway for the purists, but accrual is slow and this probably reflects the increasing unattractiveness of the oophorectomy option.

But the real point here is in danger of being missed. Oophorectomy is already a redundant treatment whether or not LHRH analogues are effective, and it is redundant because of tamoxifen. Reservations are sometimes expressed about tamoxifen in premenopausal women because it raises plasma oestradiol and does not always achieve amenorrhoea. Yet two randomised trials have shown that this is of no clinical consequence whatever: tamoxifen is as effective as oophorectomy in every respect and with minimal toxicity (Buchanan *et al.*, 1986; Ingle *et al.*, 1986). Indeed, the failure of tamoxifen to achieve postmenopausal oestrogen levels is probably a positive benefit rather than a disadvantage; unlike oophorectomy (and presumably LHR analogues) it does not appear to cause bone demineralisation, and it significantly lowers serum cholesterol and low density lipoproteins (Powles *et al.*, 1990).

The real test for the LHRH analogues is no longer against oophorectomy, but against tamoxifen. Are they more effective? Are they in any way better tolerated? How do their long term effects on bone demineralisation and plasma lipids compare? As with so many other new forms of breast cancer treatment, tamoxifen is still the one to beat.

Received 23 August 1990.

**References**

BUCHANAN, R.B., BLAMEY, R.W., DURRANT, K.R. & 6 others (1986). A randomised comparison of tamoxifen with surgical oophorectomy in premenopausal patients with advanced breast cancer. *J. Clin. Oncol.*, **4**, 1326.

CRIGHTON, I., DOWSETT, M., LAL, M. & 2 others (1989). Use of luteinising hormone-releasing hormone agonist (leuprorelin) in advanced postmenopausal breast cancer: clinical and endocrine effects. *Br. J. Cancer*, **60**, 644.

DIXON, A.R., ROBERTSON, J.F.R., JACKSON, L., NICHOLSON, R.I., WALKER, K.J. & BLAMEY, R.W. (1990). Goserelin (Zoladex) in premenopausal advanced breast cancer: duration of response and survival. *Br. J. Cancer*, **62**, 868.

EIDNE, K.A., FLANAGAN, C.A. & MILLAR, R.P. (1985). Gonadotrophin-releasing hormone binding sites in human breast carcinoma. *Science*, **229**, 989.

HARRIS, A.L., CARMICHAEL, J., CANTWELL, B.M.J. & DOWSETT, M. (1989). Zoladex: endocrine and therapeutic effects in post-menopausal breast cancer. *Br. J. Cancer*, **59**, 97.

HARVEY, H.A., LIPTON, A., MAX, D.T. & 3 others (1985). Medical castration produced by the GnRH analogue leuprolide to treat metastatic breast cancer. *J. Clin. Oncol.*, **3**, 1068.

INGLE, J.N., KROOK, J.E., GREEN, S.J. & 2 others (1986). Randomised trial of bilateral oophorectomy versus tamoxifen in premenopausal women with metastatic breast cancer. *J. Clin. Oncol.*, **4**, 178.

KAUFMANN, M., JONAT, W., KLEEBERG, U. & 11 others (1989). Goserelin, a depot gonadotrophin-releasing hormone agonist in the treatment of premenopausal patients with metastatic breast cancer. *J. Clin. Oncol.*, **7**, 1113.

KLIJN, J.G.M. (1984). Long-term LHRH-agonist treatment with metastatic breast cancer as a single treatment and in combination with other additive endocrine treatments. *Med. Oncol. & Tumor Pharmacother.*, **1**, 123.

MILLER, W.R., SCOTT, W.N., MORRIS, R. & 2 others (1985). Growth of human breast cancer cells inhibited by a luteinizing hormone-releasing hormone agonist. *Nature*, **313**, 231.

NICHOLSON, R.I. & MAYNARD, P.V. (1979). Anti-tumour activity of ICI 118630, a new potent luteinising hormone-releasing hormone agonist. *Br. J. Cancer*, **39**, 268.

PLOWMAN, P.N., NICHOLSON, R.I. & WALKER, K.J. (1986). Remissions of post-menopausal breast cancer during treatment with the luteinising hormone releasing hormone agonist ICI 118630. *Br. J. Cancer*, **54**, 903.

POWLES, T.J., TILLYER, C.R., JONES, A.L. & 4 others (1990). Prevention of breast cancer with tamoxifen – an update on the Royal Marsden Hospital pilot programme. *Eur. J. Cancer*, **26**, 680.

WAXMAN, J.H., HARLAND, S.J., COOMBES, R.C. & 4 others (1985). The treatment of postmenopausal women with advanced breast cancer with buserelin. *Cancer Chemother. Pharmacol.*, **15**, 171.

WILLIAMS, M.R., WALKER, K.J., TURKES, A. & 2 others (1986). The use of an LH-RH agonist (ICI 118630, Zoladex) in advanced premenopausal breast cancer. *Br. J. Cancer*, **53**, 629.